# Can predilatation in transcatheter aortic valve implantation be omitted? - a prospective randomized study

**DOI:** 10.1186/s13019-016-0516-x

**Published:** 2016-08-04

**Authors:** Henrik Casimir Ahn, Niels-Erik Nielsen, Jacek Baranowski

**Affiliations:** 1 Department of Cardiothoracic Surgery, University Hospital, Institution of Medical and Health Sciences, Linkoping University, Linkoping, Sweden; 2 Department of Cardiology, Linkoping University, Linkoping, Sweden; 3 Department of Physiology, Linkoping University, Linkoping, Sweden

**Keywords:** Aortic valve stenosis, Aortic valve disease, Percutaneous intervention, Balloon angioplasty

## Abstract

**Background:**

The use of a balloon expandable stent valve includes balloon predilatation of the aortic stenosis before valve deployment. The aim of the study was to see whether or not balloon predilatation is necessary in transcatheter aortic valve replacement (TAVI).

**Methods:**

Sixty consecutive TAVI patients were randomized to the standard procedure or to a protocol where balloon predilatation was omitted.

**Results:**

There were no significant differences between the groups regarding early hemodynamic results or complication rates.

**Conclusions:**

TAVI can be performed safely without balloon predilatation and with the same early results as achieved with the standard procedure including balloon predilatation. The reduction in the number of pacing periods required may be beneficial for the patient.

## Background

Transcatheter Aortic Valve Implantation (TAVI) is now a widespread procedure and there are international guidelines for selection of patients and for the reporting of results [1–3]. Long- term results have so far been convincing [4, 5] and continuous technologic and material development has improved the technique. In many centers the original TAVI protocol has been simplified over the years in an attempt to reduce or avoid any risk associated with the procedure. The standard procedure includes predilatation of the stenotic valve with a balloon before stent deployment, with at least two periods of rapid pacing per TAVI procedure. Should the balloon slide during inflation, additional periods of pacing are necessary. Some patients react with slow recovery of heart function, and we had three patients requiring resuscitation and temporary extracorporeal circulation in our first 100 TAVI patients. In 2011 we started to omit predilatation during the procedure and thereby reduced the number of pacing periods. Of 258 TAVI patients, predilatation was avoided in 139. The results indicated that the procedure could be performed safely without predilatation. As these patient groups represent two different time periods, where a learning curve could have interfered with outcome, we decided to do a prospective randomized study including all consecutive TAVI patients during the course of one year.

## Methods

Sixty consecutive TAVI patients were randomized to receive balloon predilatation or not. Valve-in-valve procedures and patients previously treated with balloon dilatation were excluded. The study was approved by the Regional Ethical Committee on Human Research, and all patients gave informed consent for participation in the study. They were randomized using a double envelope system. The sealed envelope was opened just before starting the procedure. The patients and the 1-month follow-up cardiologist performing transesophageal echocardiography were blinded to the randomization. The echocardiographic evaluation of prosthetic aortic valve regurgitation was performed by at least one cardiologist with expertise in the method. Investigations were performed in a key national lab where almost all TAVI cases at our department have been operated from the start. The VARC 2 criteria were used [3]. A bovine pericardial stent valve (Sapien XT, Edwards Inc. Santa Ana, CA) was used in all patients. Demographic data are shown in Table 1.

**Table 1:** Sixty TAVI patients in the randomized study

Patients	No balloon predilatation (*n* = 30)	Balloon predilatation (*n* = 30)	Difference between groups
Men/women (*n*)	14/16	16/14	ns
Age, mean (range)	83 (58–93)	81 (42–91)	ns
Euroscore I	17 (2–38)	19 (4–57)	ns
Euroscore II	8 (2–18)	9 (3–37)	ns
STS score	7 (1–13)	6 (2–17)	ns
PPM (*n*)	1	2	ns
Vmax m/s	4.4 (2.9–5.8)	4.3 (3.3–5.8)	ns
Mean gradient mmHg	51 (21–84)	48 (29–88)	ns
EOA cm^2^	0.6 (0.3–0.9)	0.6 (0.3–1.0)	ns

*STS* Society of Thoracic Surgeons, *PPM* Permanent pacemaker, *Vmax* maximum upstroke velocity, *EOA* effective orifice area

### Procedure

General anesthesia and transesophageal echocardiography (TOE) were routinely used. In the transfemoral group the common femoral artery was punctured in the anterior wall with ultrasound guidance. Two percutaneous closure devices (Proglide™) were inserted prior to dilatation of the vessel. Adequate cusp alignment was facilitated with ultrasound-guided placement and adjustment of the image system [6]. TOE was used to confirm correct placement of the stent valve and to evaluate the hemodynamic result.

Contrast injection in the aortic root was performed only when the fluoroscopic view or TOE was not good enough for visualizing the aortic root and cusp alignment. In the predilatation group the balloon was introduced and blown up during a period of rapid pacing (RP). If the balloon migrated, further RP was done. The stent valve was then positioned and deployed during one more pacing period. In the group without balloon dilatation, the valve was passed through the stenotic valve and the stent valve deployed during one RP period.

When the transapical approach was used we regularly passed the stenotic valve with the introducer which then was withdrawn before stent valve deployment. In the predilatation group a balloon catheter was introduced through the stenosis and blown up during RP according to the standard protocol.

### Statistics

The VARC-2 endpoint definitions were used for selected variables [3, 7, 8]. The data were prospectively saved in the hospital’s digital data-base, and also transferred to an Excel data-base for analysis. The study design and sample size were chosen with hemodynamic non-inferiority as primary endpoint. Intention-to-treat analysis of data was performed. Continuous variables were tested for normality using the Shapiro-Wilks test. Student’s *t*-test for independent samples was used to test difference between the two groups. Categorical variables were compared using the chi-square test, or Fischer’s exact test when the number of observations was very small (<5). A *p* value <0,05 between the groups was regarded as significant. Statistica (StatSoft Inc.) software was used for the calculations.

## Results

### Procedural data

Table 2 summarizes the results. There was a trend towards shorter procedure time and less contrast delivery in the no balloon dilatation (NBD) group, not reaching statistical significance. In almost half of the patients (27/60) no contrast was given. Vascular complications according to the VARC 2 [3] criteria did not differ between the groups. Pericardial bleeding at the end of the procedure occurred in 2 patients in each group. Three were successfully treated with pericardial drainage. One patient in the NBD group needed temporary circulatory support, and sternotomy and myocardial suture due to guidewire perforation. Sternotomy and myocardial suture, without ECC, was performed in another NBD patient for the same reason. One patient in the balloon dilatation (BD) group needed circulatory support over thirty minutes after the pacing period due to low output syndrome. One patient in the standard group suffered from sudden severe fall in blood pressure before pacing and balloon dilatation, and the stent valve was quickly deployed without planned balloon dilatation during one short period of pacing. In another planned BD patient the balloon migrated two times during inflation and we chose to continue without further attempts. One BD patient developed moderate valvular regurgitation, and due to clinical symptoms a valve-in-valve procedure was performed one week after the initial procedure. One patient randomized to BD had sudden onset of tachycardia with low cardiac output in association with balloon introduction through the stenosis. We avoided dilatation, and the stent valve was deployed without pacing. Valve embolization occurred in one NBD patient due to inaccurate withdrawal of the pusher before valve deployment. The valve was stented in the decending aorta and a new valve was adequately positioned and deployed. Paravalvular leakage was mild or less in all patients. There was no procedural mortality.

**Table 2:** Procedural data from the randomized study

	No balloon predilatation (*n* = 30)	Balloon predilatation (*n* = 30)	Difference between groups
Approach TF/TA (*n*)	26/4	24/6	ns
Procedure time (min)	68 (35–210)	80 (30–360)	ns
Fluoroscopy (min)	22 (6–45)	27 (9–84)	ns
No contrast (*n*)	17	10	ns
Contrast ml (*n*)	13	20	
Mean (range)	38 (20–60)	42 (20–163)	ns
Minor vasc. complic. (*n*)	2	4	ns
Major vasc. complic. (*n*)	2	1	ns
Pericardial bleeding (*n*)	2	2	ns
Sternotomi + hemostasis	2	0	ns
ECC (*n*)	1	1	ns
Crossover	0	2	ns
Valve embolization	1	0	ns
Valve dysfunction	0	1	ns
PVL = 0, (*n*)	9	5	ns
PVL median (range)	0.5 (0–1)	0.5 (0–1)	ns
No of pacing	0 pacing *n* = 1^a^	1 pacing *n* = 1^a^	Pacing:
	1 pacing *n* = 28	2 pacing *n* = 21	NBD vs BD
	2 pacing *n* = 1	3 pacing *n* = 8	*P* < 0,001

^a^Spontaneous drop in BP with Sapien™ in native valve. *TF* transfemoral, *TA* transapical, *ECC* extracorporeal circulation, *PVL* paravalvular leakage (0.5 = trace, 1 = mild), *NBD* no balloon dilatation, *BD* balloon dilatation

### One-month follow-up

Data on function did not differ between the groups. Paravalvular leak was mild or less in all patients. The number of complications was low. One patient in the NBF group received a permanent pacemaker due to bradycardia. Three patients had ischemic stroke, one disabling stroke in each group and a non-disabling stroke in the BD group. There were three deaths, one due to acute myocardial infarction in a 66 year-old man, one due cardiac arrest in a 76 year-old man, and one due to worsening heart failure before discharge in a 91 year-old man. Data are summarized in Table 3.

**Table 3:** One-month follow-up data

One-month follow- up	No balloon predilatation (*n* = 30)	Balloon predilatation (*n* = 30)	Difference between groups
Vmax m/s	2.1 (1.4–2.8)	1.9 (1.3–2.9)	ns
Mean gradient mmHg	10 (3–17)	9 (4–17)	ns
PVL = 0, (*n*)	9	3	ns
PVL median (range)	0.5 (0–1)	0.5 (0–1)	ns
Gastric bleeding	0	1	ns
Acute kidney injury (*n*)	0	0	ns
Conduction disturb. (*n*)	1	1	ns
PPM after procedure	1	0	ns
Stroke (*n*)	1	2	ns
Death (*n*)	1	2	ns

*Vmax* maximum upstroke velocity, *PVL* paravalvular leakage (0.5 = trace, 1 = mild) *PPM* permanent pacemaker

## Discussion

This study shows that it is possible to omit balloon predilatation from the standard TAVI procedure without affecting early outcome regarding hemodynamic results. This is an interesting topic that has not been well documented and yet most centers have omitted pre dilatation for the past several years. A similar conclusion has previously been reported in non-randomized series of patients using self-expandable stent valves and balloon-expandable valves [9–11]. The number of rapid pacing periods in our study was significantly reduced, and the initial and one month hemodynamic results were similar between the groups. The frequency of complications was similar and there was a trend towards shorter procedural time and less need for contrast delivery in the group without balloon predilatation. Though the study had a rather small number of patients, it was designed to investigate non-inferiority and results indicate that balloon predilatation is not necessary, but there was not the power to prove if there are advantages in outcome with this modification. We initially planned to include double the sample size in order to evaluate outcome, and planned for a 2-year study. After 1 year the members of the TAVI-team were very reluctant to proceed with the study (especially the anesthesiologists) since they considered that maintaining a protocol with at least two periods of rapid pacing and balloon pre-dilatation increased the potential risk for hemodynamic disturbances. More and more centers are beginning to avoid this maneuver to simplify the procedure, since clinical experience of avoiding predilatation has been good. Several clinical trials are ongoing but so far most centers rely on their individual experience.

Myocardial hypoperfusion and even circulatory collapse are rare but serious events after multiple RP periods [12]. TAVI has been associated with the development of acute renal failure [13–15]. Impaired renal function is often a component of comorbidity in TAVI candidates. Avoidance of one period of rapid pacing, by avoiding balloon predilatation, shortens the procedure and reduces the risk for temporary circulatory failure. In patients at risk for renal failure it would seem beneficial to omit unnecessary maneuvers that could lead to temporary renal hypoperfusion. It is reasonable to believe that the modified procedure with just one RP period should reduce this risk. The potential benefits of avoiding use of the predilatation balloon include less manipulation of the aorta and the LV outflow tract. Theoretically this should reduce the risk of emboli and conduction disturbances. If the balloon is used for gaging size or for other purposes then this modified procedure clearly has a drawback. We have now used it routinely in over 200 patients and have had two cases where we had real problems passing the native valve, and had to use a lasso wire to support the nosecone. In 1 case we had to pre-dilate before the stent valve passed the native valve. Expansion of the stent has never been a problem.

In this series of 60 patients we had two patients requiring emergency cardiac surgery for pericardial bleeding due to guidewire perforation of the left ventricle. In one further patient we had to use temporary ECC to support the patient while suturing the perforation. Three major vascular complications needed endovascular intervention. These six patients all recovered and were survivors at the 1-month follow-up illustrating the importance of heart team cooperation during TAVI.

Based on our experience with guidewire perforation, we have replaced the stiff guidewire with a soft wire through the delivery system when reaching the ascending aorta [16].

This atraumatic wire can be used when balloon predilatation is avoided. The soft wire is passed through the stenosis into the left ventricle and is used as support for final positioning of the stent valve. More than one hundred patients have been treated this way without any pericardial bleeding [16].

### Limitation and strength

The relatively small sample size does not have the power to analyse long-term clinical outcome. The strength of the study is its design, where all patients over a period of one year coming for TAVI due to native AS were included and randomized to balloon pre-dilatation or not.

## Conclusion

TAVI can be performed safely without balloon predilatation and without compromising the early hemodynamic outcome of the procedure. The reduction in the number of pacing periods required may be beneficial for the patient.
